# Supplementary material for: Electroencephalographic derived network differences in Lewy body dementia compared to Alzheimer’s disease patients
Source: Sci Rep. 2018 Mar 15;8:4637. doi: 10.1038/s41598-018-22984-5 (PMC5854590; doi:10.1038/s41598-018-22984-5)
Supplement: Supplementary file 1 — Supplementary Information [file 41598_2018_22984_MOESM1_ESM.docx]

Supplementary Material

Electroencephalographic derived network differences in Lewy body dementia compared to Alzheimer’s disease patients

Authors:

Authors: Luis R. Peraza^1,*^, Ruth Cromarty^1^, Xenia Kobeleva^2^, Michael J. Firbank^1^, Alison Killen^1^, Sara Graziadio^3^, Alan J. Thomas^1^, John T. O’Brien^4,1^, John-Paul Taylor^1^

1. Institute of Neuroscience, Campus for Ageing and Vitality, Newcastle University, NE4 5PL United Kingdom.
2. University Hospital Bonn, Clinic for Neurology, 53127, Bonn, Germany
3. NIHR Newcastle In Vitro Diagnostics Co-operative, Newcastle University, Newcastle upon Tyne, NE2 4HH, United Kingdom
4. Department of Psychiatry, University of Cambridge School of Medicine, Cambridge, CB2 0SP, United Kingdom

Corresponding author:

*Luis R. Peraza

Biomedical Research Building 3^rd^ floor,

Campus for Ageing and Vitality

Newcastle University, NE45PL

Newcastle upon Tyne, UK.

Tel: +44 0191 208 1125

Email: luis.peraza-rodriguez@newcastle.ac.uk

**
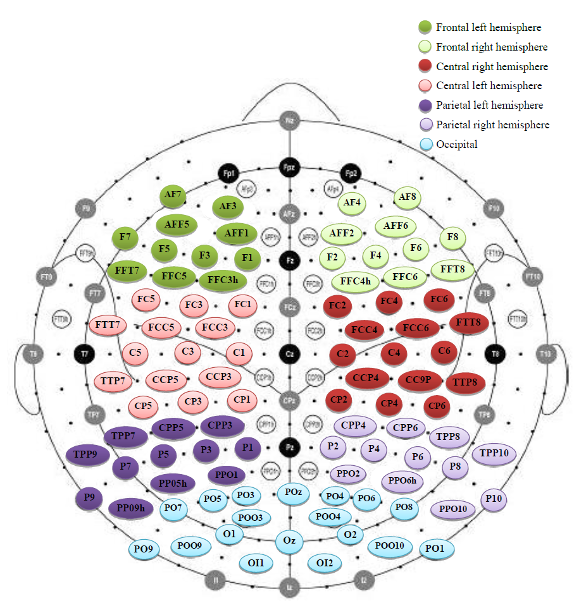
**

**Supplementary Figure S1**. The 10-5 EEG system layout. The occipital electrodes used for the estimation of dominant frequency (DF) and its variability (DFV) are shown in blue colour with PO and O prefixes.

**Supplementary Table S1**. Two-way ANOVA results for the effects of group (HC, AD, DLB and PDD), frequency (delta, theta, high-theta, alpha, beta and dominant frequency) and the interaction. P-values shown adjusted for multiple tests –i.e. multiplied by the Bonferroni factor. PLI height (SD) was not significant after Bonferroni correction.

| **MST parameter** | **Two-Way ANOVA effect** | **Effect p-value** |
| --- | --- | --- |
| Degree_max_ (mean) | group | 0.026 |
| Leaf ratio (mean) | group | 0.018 |
| Diameter (mean) | group | <0.001 |
| Eccentricity (mean) | group | <0.001 |
| Radius (mean) | group | <0.001 |
| PLI mean (mean) | group*frequency | <0.001 |
| PLI mean (SD) | group*frequency | <0.001 |
| PLI leaf (mean) | group*frequency | <0.001 |
| PLI leaf (SD) | group*frequency | <0.001 |
| PLI root (mean) | group*frequency | <0.001 |
| PLI root (SD) | group*frequency | <0.001 |
| PLI height (mean) | group*frequency | <0.001 |

**Supplementary Table S2**. Global effects of group diagnosis; two-way ANOVA tests.

|  | **HC** | **AD** | **DLB** | **PDD** |
| --- | --- | --- | --- | --- |
| Degree_max_ | 18.12 (3.64) | 17.13 (2.22) | 17.05 (2.23) | 17.44 (2.37) |
| Leaf ratio | 0.653 (0.03) | 0.643 (0.02) | 0.644 (0.02) | 0.648 (0.02) |
| Diameter | 17.02 (1.00) | 17.32 (1.05) | 17.44 (0.96) | 17.46 (1.04) |
| Eccentricity | 13.08 (0.78) | 13.31 (0.82) | 13.40 (0.75) | 13.43 (0.82) |
| Radius | 8.76 (0.50) | 8.90 (0.53) | 8.98 (0.48) | - 1. 0.53) |

**Supplementary Table S3**. Acronyms and meanings of network measures and other variables.

| **Acronym** | **Meaning** |
| --- | --- |
| PLI | Phase lag index |
| MST | Minimum Spanning Tree |
| SD | Standard Deviation (Measure of variability) |
| DF | Dominant Frequency |
| DFV | Dominant Frequency Variability |
| PLI band mean/SD | Average PLI across the entire MST network for the indicated frequency band; mean/SD values throughout the 50-second segment |
| PLI root band mean/SD | Average PLI for the root node in the MST network for the indicated frequency band; mean/SD values throughout the 50-second segment |
| PLI leaf band mean/SD | Average PLI for the leaf nodes in the MST network for the indicated frequency band; mean/SD values throughout the 50 -second segment |
| PLI height band mean/SD | Difference between average PLI values between the root and the leaves in the MST network; mean/SD values throughout the 50-second segment |

**Supplementary Table S4**. Minimum spanning tree (MST) measures. Mean and standard deviation (SD), Significant one-way four-group ANOVAs with p-value < 0.05, Bonferroni corrected for multiple tests.

|  | **HC** | | **AD** | | **DLB** | | **PDD** | |
| --- | --- | --- | --- | --- | --- | --- | --- | --- |
| **MST parameter** | **Mean** | **SD** | **Mean** | **SD** | **Mean** | **SD** | **Mean** | **SD** |
| Diameter High-theta mean | 17.3493 | 0.9692 | 17.9443 | 0.6238 | 18.3624 | 0.8206 | 18.5637 | 0.6481 |
| Eccentricity High-theta mean | 13.3330 | 0.7645 | 13.7930 | 0.4905 | 14.1187 | 0.6369 | 14.2904 | 0.5033 |
| Radius High-theta mean | 8.9256 | 0.4841 | 9.2214 | 0.3149 | 9.4392 | 0.4135 | 9.5287 | 0.3344 |
| PLI Theta mean | 0.6662 | 0.0285 | 0.7074 | 0.0326 | 0.7095 | 0.0436 | 0.7241 | 0.0257 |
| PLI High-theta mean | 0.6984 | 0.0483 | 0.7289 | 0.0433 | 0.7638 | 0.0406 | 0.7638 | 0.0372 |
| PLI Alpha mean | 0.6403 | 0.0736 | 0.5733 | 0.0359 | 0.5701 | 0.0432 | 0.5324 | 0.0275 |
| PLI Alpha SD | 0.0831 | 0.0203 | 0.0659 | 0.0147 | 0.0629 | 0.0111 | 0.0568 | 0.0078 |
| PLI Beta mean | 0.3369 | 0.0200 | 0.3285 | 0.0165 | 0.3120 | 0.0202 | 0.3086 | 0.0182 |
| Leaf Theta mean | 0.2352 | 0.0105 | 0.2587 | 0.0202 | 0.2660 | 0.0329 | 0.2757 | 0.0209 |
| Leaf Theta SD | 0.0437 | 0.0437 | 0.0529 | 0.0529 | 0.0577 | 0.0577 | 0.0621 | 0.0621 |
| Leaf High-theta mean | 0.2670 | 0.0323 | 0.2811 | 0.0373 | 0.3065 | 0.0319 | 0.3077 | 0.0307 |
| Leaf Alpha mean | 0.2566 | 0.0529 | 0.2086 | 0.0210 | 0.2108 | 0.0216 | 0.1923 | 0.0130 |
| Leaf Alpha SD | 0.0684 | 0.0247 | 0.0473 | 0.0108 | 0.0449 | 0.0095 | 0.0396 | 0.0075 |
| Leaf Beta mean | 0.1164 | 0.0120 | 0.1099 | 0.0080 | 0.1044 | 0.0098 | 0.1028 | 0.0068 |
| Root Theta mean | 0.7059 | 0.0301 | 0.7504 | 0.0322 | 0.7496 | 0.0378 | 0.7647 | 0.0255 |
| Root High-theta mean | 0.7347 | 0.0498 | 0.7680 | 0.0386 | 0.7981 | 0.0366 | 0.7957 | 0.0351 |
| Root Alpha mean | 0.6836 | 0.0736 | 0.6151 | 0.0387 | 0.6140 | 0.0469 | 0.5751 | 0.0330 |
| Root Alpha SD | 0.1008 | 0.0207 | 0.0852 | 0.0154 | 0.0805 | 0.0117 | 0.0782 | 0.0083 |
| Root Beta mean | 0.3643 | 0.0233 | 0.3547 | 0.0194 | 0.3369 | 0.0217 | 0.3340 | 0.0215 |
| Height High-theta mean | 0.4678 | 0.0304 | 0.4878 | 0.0163 | 0.4916 | 0.0111 | 0.4880 | 0.0134 |
| Height Alpha mean | 0.4270 | 0.0268 | 0.4064 | 0.0202 | 0.4032 | 0.0277 | 0.3828 | 0.0235 |
| Height Beta mean | 0.2480 | 0.0142 | 0.2448 | 0.0127 | 0.2325 | 0.0127 | 0.2312 | 0.0166 |

**Supplementary Table S5**. Regression model: Clinical variable = β_1_*Network variable + β_2_*D_DLB_ + β_3_*D_PDD_ + Intercept

| **Network measure** | **Clinical variable** | **Network variable** | | **DLB variable** | | **PDD variable** | | **Linear regression** | |
| --- | --- | --- | --- | --- | --- | --- | --- | --- | --- |
|  |  | *β*_1_ | p-value | *β*_2_ | p-value | *β*_3_ | p-value | p-value | *R^2^* |
| PL high-theta mean | NPI hallucinations | -9.875 | 0.046 | 2.082 | 7.64E-05 | 2.894 | 3.86E-07 | 4.729E-06 | 0.334 |
| PLI theta mean | Animal naming | -31.466 | 0.040 | 0.071 | 0.954 | 0.704 | 0.593 | 0.231 | 0.061 |
| PLI theta mean | CAMCOG memory | -39.831 | 0.020 | 7.238 | 1.5E-06 | 7.256 | 5.05E-06 | 9.035E-07 | 0.363 |
| PLI theta mean | CAMCOG total | -119.695 | 0.015 | 9.790 | 0.015 | 8.838 | 0.039 | 0.009 | 0.155 |
| Leaf theta mean | Animal naming | -48.141 | 0.022 | 0.358 | 0.772 | 0.998 | 0.454 | 0.149 | 0.075 |
| Leaf theta mean | CAMCOG memory | -58.912 | 0.013 | 7.586 | 0.000 | 7.593 | 2.62E-06 | 5.93E-07 | 0.371 |
| Leaf theta mean | CAMCOG total | -169.770 | 0.012 | 10.783 | 0.008 | 9.728 | 0.026 | 0.008 | 0.159 |
| Root theta mean | CAMCOG memory | -43.243 | 0.020 | 7.120 | 2.06E-06 | 7.210 | 5.36E-06 | 8.75E-07 | 0.364 |
| Root theta mean | CAMCOG total | -126.377 | 0.018 | 9.439 | 0.019 | 8.651 | 0.043 | 0.011 | 0.151 |
| Dominant frequency | Animal naming | 1.364 | 0.013 | 1.143 | 0.379 | 1.963 | 0.180 | 0.099 | 0.088 |
| Dominant frequency | CAMCOG attention | 0.587 | 0.032 | 1.082 | 0.099 | 1.150 | 0.117 | 0.133 | 0.078 |
| Dominant frequency | CAMCOG executive | 1.542 | 0.007 | -0.057 | 0.966 | 0.458 | 0.761 | 0.030 | 0.122 |
| Dominant frequency | CAMCOG total | 5.243 | 0.003 | 13.910 | 0.001 | 13.696 | 0.004 | 0.002 | 0.191 |
| Dominant frequency | MMSE | 1.193 | 0.029 | 3.557 | 0.008 | 4.197 | 0.005 | 0.017 | 0.138 |
| Dominant frequency | Trail A | -21.883 | 0.027 | 7.998 | 0.731 | 60.946 | 0.025 | 4.18E-04 | 0.252 |
| Dominant frequency | Verbal fluency | 6.928 | 3.7E-05 | -0.120 | 0.975 | 4.293 | 0.318 | 1.71E-04 | 0.256 |

**Supplementary Table S6**. Network measures and dominant frequency variables used for the logistic classification; three diagnosis scenarios.

| Diagnostic scenario | Network and frequency measures  used in the logistic regression |
| --- | --- |
| DLB vs AD | PLI high-theta mean |
|  | PLI beta mean |
|  | PLI beta mean |
|  | PLI leaf high-theta mean |
|  | PLI root high-theta mean |
|  | PLI root beta mean |
|  | PLI height beta mean |
|  | DF |
|  | DFV |
| DLB vs PDD | PLI alpha mean |
|  | PLI root alpha mean |
|  | PLI height alpha mean |
| HC vs AD+DLB | PLI theta mean |
|  | PLI alpha mean |
|  | PLI alpha SD |
|  | PLI leaf theta mean |
|  | PLI leaf alpha mean |
|  | PLI leaf alpha SD |
|  | PLI root theta mean |
|  | PLI root high-theta mean |
|  | PLI root alpha mean |
|  | PLI root alpha SD |
|  | PLI height alpha mean |
|  | DF |

**Supplementary Table S7**. Multivariate regression for the Levodopa equivalent daily dose (LEDD) effect in the EEG features from Lewy body disease participants. A dichotomous variable D_1_ is included to account for the group effect (DLB, PDD).

Model: LEDD ~ B_1_*EEG feature + B_2_*D_1_ + Intercept.

| Network variable / EEG feature | *B_1_* LEDD | p-value | *B_2_* group | p-value  1x10^-6 | *R^2^* |
| --- | --- | --- | --- | --- | --- |
| PLI high-theta mean | 0.2732 | 0.2732 | 653.2946 | 0.0074 | 0.5503 |
| PLI beta mean | 0.1659 | 0.1659 | 641.9668 | 0.01 | 0.5578 |
| Leaf high-theta mean | 0.4697 | 0.4697 | 652.067 | 0.0094 | 0.543 |
| Root high-theta mean | 0.2512 | 0.2512 | 656.9878 | 0.0063 | 0.5515 |
| Root beta mean | 0.1969 | 0.1969 | 645.2121 | 0.0092 | 0.5552 |
| Height beta mean | 0.2785 | 0.2785 | 648.6862 | 0.009 | 0.55 |
| PLI alpha mean | 0.8681 | 0.8681 | 645.33 | 0.1902 | 0.5377 |
| Root alpha mean | 0.7332 | 0.7332 | 638.1485 | 0.1686 | 0.5386 |
| Height alpha mean | 0.594 | 0.594 | 633.4922 | 0.1022 | 0.5405 |
| PLI theta mean | 0.6548 | 0.6548 | 644.9183 | 0.0211 | 0.5395 |
| PLI alpha std | 0.8345 | 0.8345 | 659.2042 | 0.0226 | 0.5379 |
| Leaf theta mean | 0.5935 | 0.5935 | 644.6637 | 0.0185 | 0.5405 |
| Leaf alpha mean | 0.9883 | 0.9883 | 652.6527 | 0.146 | 0.5374 |
| Leaf alpha std | 0.6557 | 0.6557 | 640.535 | 0.0437 | 0.5395 |
| Root theta mean | 0.7471 | 0.7471 | 646.322 | 0.0239 | 0.5385 |
| Root alpha std | 0.8242 | 0.8242 | 655.1886 | 0.0104 | 0.5379 |
| DF | 0.2186 | 0.2186 | 688.8197 | 0.0058 | 0.5536 |
| DFV | 0.1049 | 0.1049 | 652.4101 | 0.0051 | 0.5651 |
| Height high-theta mean | 0.1279 | 0.1279 | 674.375 | 0.0033 | 0.5619 |
